# Reduced *γ*-Aminobutyric Acid and Glutamate+Glutamine Levels in Drug-Naïve Patients with First-Episode Schizophrenia but Not in Those at Ultrahigh Risk

**DOI:** 10.1155/2016/3915703

**Published:** 2016-11-28

**Authors:** Junjie Wang, Yingying Tang, Tianhong Zhang, Huiru Cui, Lihua Xu, Botao Zeng, Yu Li, Gaiying Li, Chunbo Li, Hui Liu, Zheng Lu, Jianye Zhang, Jijun Wang

**Affiliations:** ^1^Shanghai Key Laboratory of Psychotic Disorders, Shanghai Mental Health Center, Shanghai Jiaotong University School of Medicine, Shanghai 200030, China; ^2^Shanghai Key Laboratory of Magnetic Resonance and Department of Physics, East China Normal University, Shanghai 200062, China; ^3^Bio-X Institutes, Key Laboratory for the Genetics of Developmental and Neuropsychiatric Disorders (Ministry of Education), Shanghai Jiaotong University, Shanghai 200030, China; ^4^MR Collaboration, Siemens Healthcare Ltd., Shanghai, China; ^5^Department of Psychiatry, Shanghai Tongji Hospital, Tongji University School of Medicine, Shanghai 200065, China; ^6^Department of Radiology, Shanghai Mental Health Center, Shanghai Jiaotong University School of Medicine, Shanghai 200030, China

## Abstract

Altered *γ*-aminobutyric acid (GABA), glutamate (Glu) levels, and an imbalance between GABAergic and glutamatergic neurotransmissions have been involved in the pathophysiology of schizophrenia. However, it remains unclear how these abnormalities impact the onset and course of psychosis. In the present study, 21 drug-naïve subjects at ultrahigh risk for psychosis (UHR), 16 drug-naïve patients with first-episode schizophrenia (FES), and 23 healthy controls (HC) were enrolled. In vivo GABA and glutamate+glutamine (Glx) levels in the medial prefrontal cortex were measured using proton magnetic resonance spectroscopy. Medial prefrontal GABA and Glx levels in FES patients were significantly lower than those in HC and UHR, respectively. GABA and Glx levels in UHR were comparable with those in HC. In each group, there was a positive correlation between GABA and Glx levels. Reduced medial prefrontal GABA and Glx levels thus may play an important role in the early stages of schizophrenia.

## 1. Introduction

Schizophrenia is a severe psychiatric disorder that affects 1% of the population worldwide; it is characterized by a complex phenotype, including positive, negative, affective symptoms and cognitive impairments [1, 2]. Schizophrenia psychosis usually occurs in late adolescence and early adulthood between ages of 18 and 25, a key period for the maturation of the prefrontal cortex [3]. A recent neurodevelopmental model of schizophrenia has proposed that an imbalance of excitation and inhibition in the prefrontal cortex is involved in the pathophysiology of schizophrenia [4]. Data from human and nonhuman primate brains have provided evidence of N-methyl-D-aspartate (NMDA) receptor hypofunction in schizophrenia, contributing to the excitation of pyramidal neurons indirectly by reducing the activity of inhibitory *γ*-aminobutyric acid (GABA)ergic interneurons [4]. The activity of GABA interneurons is mediated by NMDA receptors, while glutamate (Glu) serves as a precursor for the synthesis of GABA and glutathione [5–8]. GABA interneurons, in turn, project to pyramidal neurons and influence their excitation [9–11]. Postmodern studies have further shown reduced synapse density in glutamatergic pyramid cells in schizophrenia, linking changes of synaptic plasticity to excitation-inhibition imbalance [4]. Excessive excitatory synaptic pruning in the prefrontal cortex may cause progressive brain tissue loss and lead to the development of psychosis [12]. GABA activity may be decreased in certain brain regions in schizophrenia, which may lead to reduce cortical plasticity and abnormal pruning [13]. Using advanced proton magnetic resonance spectroscopy (^1^H MRS), neurochemical concentrations such as glutamate and *γ*-aminobutyric acid can be measured in vivo [12, 14–16]. There is increasing evidence from MRS studies linking glutamatergic or GABAergic disturbances to cognitive deficits and the pathophysiology of schizophrenia [15, 17–21]. Thus, studies on the imbalance between GABAergic and glutamatergic systems should shed light into uncovering the mechanisms of schizophrenia [11, 22].

Aberrant GABAergic or glutamatergic levels in schizophrenia have been separately reported in previous studies [18, 21, 23–31]. Meta-analyses focusing on ^1^H MRS studies have shown glutamate alterations across several brain regions in schizophrenia [12, 16]. Marsman et al. reported lower Glu and higher glutamine (Gln) levels in the medial frontal cortex in schizophrenia [12]. Merritt et al. further suggested higher glutmate+glutamine (Glx) levels of the medial frontal cortex in subjects at high risk for schizophrenia rather than first-episode or chronic schizophrenia [16]. Higher Glu and Glx levels in the basal ganglia, Gln in the thalamus, and Glx levels in the medial temporal lobe were also found in schizophrenia [16]. Reduced Glx or Glu levels in the medial prefrontal cortex (mPFC) and anterior cingulate cortex (ACC) were observed in several studies on chronic schizophrenia but could not be repeated by other studies [30, 32, 33]. The mixed findings on glutamatergic alterations could be due to the illness progress, severity, duration, or pharmacological treatment [22]. Further studies on the ultrahigh risk for psychosis (UHR) and first-episode schizophrenia (FES), in particular, including drug-naïve subjects, are necessary to exclude the effects of medications.

In vivo GABA measurements have recently become available, aided by newly developed MRS sequences, Meshcher-Garwood point resolved spectroscopy (MEGA-PRESS), and J-Point resolved spectroscopy (JPRESS) [34, 35]. GABA alterations in schizophrenia are also controversial [15, 21, 27, 28, 36–39]. Several studies have reported lower GABA levels in the ACC, bilateral calcarine sulci, and mPFC in chronic schizophrenia [15, 38, 39]. However, unchanged or elevated GABA levels in the ACC and mPFC have also been observed [15, 28, 37, 39]. Evidence of GABA alterations in first-episode schizophrenia remains limited [22]. Three studies on drug-naïve or medicine-free FES patients showed reduced GABA levels in bilateral calcarine sulci and the left basal ganglia [27, 36]. In addition, a recent UHR study showed higher GABA in the dorsal caudate and mPFC [17]. Therefore, more studies on GABA alterations in both drug-naïve UHR and FES are needed.

One possible reason for the controversial findings of either GABA or Glu alterations in schizophrenia could be that these two neurotransmitter systems interact with each other [22, 40–43]. The changes in GABA or Glu in schizophrenia should not be determined separately [44]. Recently, several researchers have begun to examine the correlations between GABA and glutamatergic metabolites [17, 19]. Kegeles et al. found a strong positive correlation between medial prefrontal GABA and Glx levels across chronic schizophrenia and healthy controls (HC) [19]. Similarly, de la Fuente-Sandoval et al. also suggested significant correlations between these two neurotransmitters in both the mPFC and dorsal caudate across UHR and HC [17]. In addition, these correlations could differ among various brain regions in the two groups. For example, UHR subjects had a positive correlation between GABA and Glx levels in the mPFC rather than the caudate, while HC had a positive correlation between GABA and Glx in the caudate rather than the mPFC [17]. These results suggested that correlations between GABA and Glx could be an important feature for dysfunctional GABAergic and glutamatergic neurotransmitters. Thus, more attention should be paid to studies combining the GABA and Glu alterations in both drug-naïve FES patients and UHR subjects. This holds great promise for uncovering the impact of GABA and Glu alterations on the onset and course of psychosis.

In the present study, we recruited drug-naïve UHR subjects, drug-naïve FES patients, and HC subjects and measured both GABA and Glx levels in the mPFC. The mPFC was selected as the region of interest because it has been widely implicated in schizophrenia, including by the postmortem [45] and MRS studies mentioned [17, 19, 46]. We hypothesized that (1) medial prefrontal GABA or Glx levels should change in gradient with illness progression, and these changes would be more prominent in FES patients than in UHR subjects; (2) GABA levels should be correlated with Glx levels in FES and UHR subjects, and the correlations may differ across the three groups.

## 2. Materials and Methods

### 2.1. Subjects

#### 2.1.1. UHR Group

Twenty-one UHR individuals were recruited from the outpatients in Shanghai Mental Health Center (SMHC). All UHR subjects met the criteria for at-risk mental state, as defined by the Structured Interview for Prodromal Syndromes (SIPS) and Scale of Prodromal Syndromes (SOPS) [47, 48]. The method for identifying UHR subjects with prodromal symptoms was introduced in detail in our previous study [48]. All UHR subjects were drug-naïve and completed their assessments on the day of their first visit to SMHC. Other inclusion criteria were being between the ages of 16 and 40 years and having at least nine years of education. Exclusion criteria included current pregnancy, major medical or neurological illness, or a history of suicide risk or alcohol or drug abuse. The mean duration of prodromal symptoms in the UHR group was 10.93 ± 16.40 months. Written informed consent was obtained from each UHR subject. Written informed consent was also obtained from his/her legal guardian if the UHR subject was younger than 18 years old.

#### 2.1.2. FES Group

Sixteen FES patients were recruited from SMHC and met the diagnostic criteria for schizophrenia or schizophreniform psychosis based on the Structured Clinical Interview for DSM-IV (patient edition) [49]. These FES patients visited our hospital seeking help for the first time upon being diagnosed with schizophrenia and recruited, and they completed their assessments on the day of their first visit to SMHC. Inclusion criteria for FES patients were having a first-episode illness, no history of exposure to antipsychotics, being between the ages of 16 and 40 years, and having at least nine years of education. Exclusion criteria included a current pregnancy, major medical or neurological illness, or a history of suicide risk or alcohol or drug abuse. Clinical symptoms of each FES patient were assessed using the 24-item Brief Psychiatric Rating Scale (BPRS, expanded version) [50, 51]. The mean duration of psychosis in the FES group was 6.94 ± 6.03 months. Written informed consent was obtained from each FES subject. Written informed consent was also obtained from his/her legal guardian if the FES subject was younger than 18 years old.

#### 2.1.3. HC Group

Twenty-three HCs were recruited by advertisement. All subjects were given the SCID (nonpatient edition) [52]. None had a history of psychiatric illnesses or a family history of mental disorders. Other inclusion criteria were being between the ages of 16 and 40 years and having at least nine years of education. Exclusion criteria included a current pregnancy, major medical or neurological illness, and alcohol or drug abuse. Written informed consent was obtained from each HC subject. Written informed consent was also obtained from his/her legal guardian, if the HC subject was younger than 18 years old.

The study was approved by the Research Ethics Committee of SMHC and in accordance with the Declaration of Helsinki. Handedness was determined by self-report from each participant, and all subjects were right-handed (see Table 1). None of the participants had any contraindication for magnetic resonance imaging (MRI).

### 2.2. MRI Acquisition

MRI data were obtained using a 3-Tesla Siemens Verio MR scanner with a 32-channel head coil (Siemens AG, Erlangen, Germany). The head position was fixed with foam padding to minimize movement artifacts.

Anatomical T1-weighted images were acquired using a three-dimensional magnetic preparation fast gradient echo (3D-MRPAGE) sequence with echo time (TE) = 2.96 ms, repetition time (TR) = 2300 ms, field of view (FOV) = 240 × 240 mm^2^, 256 × 256 matrix, a slice thickness of 1.0 mm, and 192 continuous sagittal slices. The T1-weighted images were used to localize the volume-of-interest (VOI) for the following MRS acquisition. All scans were reviewed by a senior radiologist who evaluated whether there were obvious artifacts, signal losses, or gross pathology.

**Figure 1:**
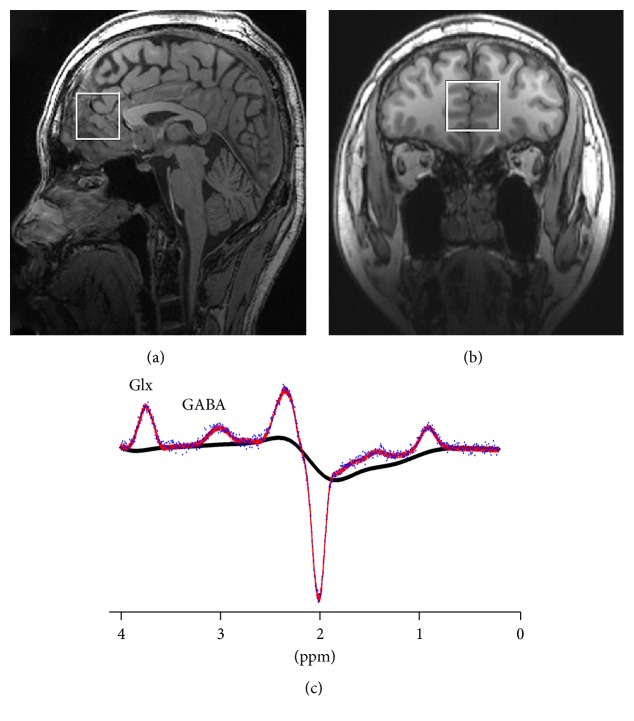
Midsagittal (a) and axial (b) views of the location of the 30 × 30 × 30 mm^3^ voxel (white square) in the mPFC. (c) Representative MRS spectrum of GABA and Glx fitted by the LCModel from one HC. The blue line is the raw experimental spectrum. The red line is the model-fitting of the experimental spectrum. The black line is the background signal. The peaks of GABA and Glx are shown.

The MRS data were acquired using a MEGA-PRESS sequence with TR = 1500 ms, TE = 68 ms, and 128 averages with water suppression. The VOI (30 × 30 × 30 mm^3^) of the mPFC was localized in the midsagittal and coronal slices, as shown in Figure 1. The VOI included Brodmann areas 24 and 32 (containing part of the anterior cingulate cortex). Six orthogonal fat saturation bands were placed surrounding the VOI to avoid signal interference. Automated shimming followed by manual shimming was conducted to reduce the water signal full-width at half maximum (FWHM) below 25 Hz.

### 2.3. Spectrum Quantification

MRS raw data were processed using the LCModel software (version 6.3-0I) [53]. Absolute GABA and Glx concentrations were quantified using scaling correction, the correction for relaxation, and partial volume effects based on the LCModel package and LCMgui, respectively. The edited spectra were fit using LCM-basis functions that were generated from phantom measurements using the MEGA-PRESS sequence with the appropriate acquisition parameters [54]. The GABA peak arose at 3.01 ppm and the Glx peak at 3.74 ppm [22]. The criteria for selecting reliable metabolite concentrations were based on the % SD of the fit for each metabolite, reflecting the Cramer–Rao lower bounds (CRLB) for the LCModel analysis [28, 55, 56]. Only the results with the % SD below 20% were included in the following analysis [56]. We obtained high spectral quality, as the % SD of all spectra from the three groups was lower than 15%.

### 2.4. Tissue Segmentation

To calculate the proportion of grey matter (GM), white matter (WM), and cerebrospinal (CSF) contained in the VOI, the volumetric 3D MPRAGE MRI data were segmented using the SPM8 software. In-house software developed in MATLAB (MathWorks Natick, MA) was then implemented to create a segmentation mask for each voxel, from which the proportions of GM, WM, and CSF were calculated. To obtain the tissue-composition-corrected metabolite intensities, each metabolite value was corrected for the CSF content of the VOI using the following formula: corrected metabolite level = uncorrected metabolite level/(1 − *C*), where *C* is the fractional CSF content of the VOI [30].

### 2.5. Statistical Analysis

All statistical analyses were conducted using SPSS v.20.0. The normality for the distribution of all independent variables, including age, years of education, and metabolite concentrations, was examined using Kolmogorov-Smirnov tests. Demographic variables, GM, WM, and CSF volumes within the VOI were compared among the three groups using analysis of variance (ANOVA). Chi-squared tests were performed to assess the group effect on gender and handedness. One-way ANOVA with the factor of group (UHR, FES, and HC) was conducted to compare group differences on GABA and Glx levels.* Post hoc* tests were performed between each two groups with Bonferroni corrections for multiple comparisons (*P* = 0.017 was the significant threshold level).

## 3. Results

### 3.1. Demographic and Clinical Characteristics

Demographic and clinical characteristics for three groups are shown in Table 1. All demographic variables, including age and years of education, were normally distributed. Age, education, gender, and handedness were all matched among the three groups. There was a significant group difference in tobacco use.

### 3.2. Voxel Tissue Composition, Spectral Quality, and Metabolite Levels

No data were discarded due to poor quality. These three groups did not differ in their signal-to-noise ratio (SNR) (*P* = 0.65) and FWHM (*P* = 0.97) from the LCModel. No between-group differences were found in the proportion of GM (*P* = 0.83), WM (*P* = 0.85), or CSF (*P* = 0.81) in the VOI of mPFC (shown in Table 2).

**Figure 2:**
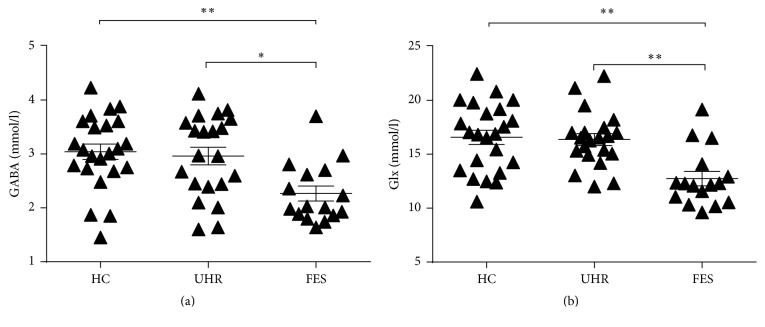
Absolute concentrations of GABA (a) and Glx (b) among FES patients and UHR and HC subjects. Lower GABA and Glx concentrations were observed in FES patients than UHR and HC subjects (*∗* for *P* < 0.05 and *∗∗* for *P* < 0.01).

There were significant main group effects on both GABA (*P* = 0.002) and Glx (*P* < 0.001) levels.* Post hoc tests* demonstrated significantly lower GABA levels in FES patients (2.27 ± 0.56 mmol/l) than HC (3.04 ± 0.69 mmol/l, *P* = 0.003) and UHR participants (2.96 ± 0.75 mmol/l, *P* = 0.01).* Post hoc tests *also revealed significantly lower Glx levels in FES patients (12.75 ± 2.66 mmol/l) compared with HC (16.57 ± 3.17 mmol/l, *P* < 0.001) and UHR subjects (16.37 ± 2.56 mmol/l, *P* = 0.001). However, both GABA and Glx levels in UHR subjects were comparable with HC subjects (*P* = 1.00 for GABA, *P* = 1.00 for Glx) (shown in Figure 2). Removing the four smoking HC subjects did not change the presence or absence of statistical significance.

**Figure 3:**
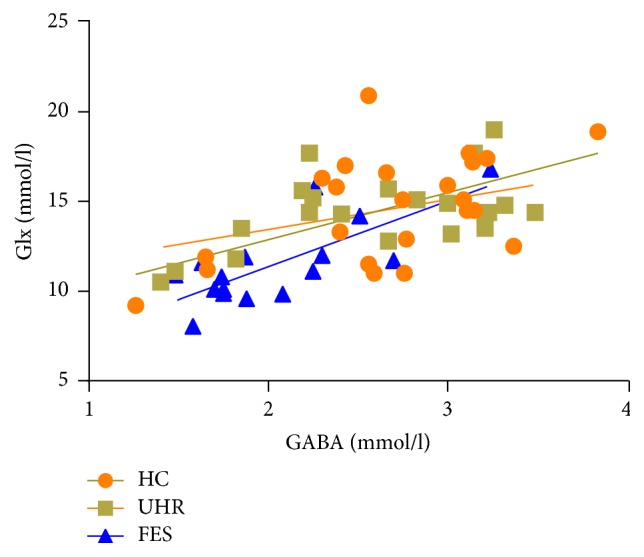
Scatter plots with linear regression fit for the relationship between GABA and glutamate+glutamine (Glx) levels among patients with first-episode schizophrenia (FES, *r* = 0.79, *P* < 0.001), subjects at ultrahigh risk for psychosis (UHR, *r* = 0.56, *P* = 0.008), and healthy controls (HC, *r* = 0.53, *P* = 0.009).

There were positive correlations between GABA and Glx levels in each group (*r* = 0.53, *P* = 0.009 for HC; *r* = 0.56, *P* = 0.008 for UHR; *r* = 0.79, *P* < 0.001 for FES), as shown in Figure 3. Further direct comparison of the correlation coefficients with *z*-transformation between the three groups did not indicate any significant differences (*z* = −1.35, *P* = 0.18 for HC* versus* FES; *z* = 0.13, *P* = 0.89 for HC* versus* UHR; *z* = −1.21, *P* = 0.23 for UHR* versus* FES). In addition, the Glx/GABA ratio did not indicate a significant main group effect (*F* = 0.11, *P* = 0.90; 5.63 ± 1.21 for HC, 5.79 ± 1.24 for UHR; 5.73 ± 0.88 for FES).

## 4. Discussion

The present study measured both GABA and Glx concentrations among drug-naïve UHR subjects, drug-naïve FES patients, and HC subjects. Significantly lower GABA and Glx levels in the mPFC were observed in drug-naïve FES patients than UHR or HC subjects, while GABA and Glx levels in UHR subjects were comparable with HC subjects. In addition, GABA and Glx levels were positively correlated within each group. Our findings, which are not confounded by medications, suggested that reduced GABA and Glx levels should play an important role in the early stages of psychosis.

Reduced medial prefrontal GABA concentration in FES patients indicates the existence of a dysfunctional GABA neurotransmitter system in the early stages of schizophrenia. In vivo measurements of GABA concentrations have recently become available by advanced MRS sequences [34]. However, findings showing GABA alterations in FES patients are relatively limited and less consistent [27, 36]. Kelemen et al. found lower GABA/Cr levels in the bilateral calcarine sulci in drug-naïve FES patients [27]. The GABA reduction remained after taking antipsychotic medication for eight weeks [27]. Lower GABA/Cr ratios were also found in the left basal ganglia in early-onset FES and remained after six months of treatment with antipsychotic medications in another study [36]. However, unchanged GABA/Cr ratios have been revealed in both the frontal lobe and parietooccipital lobe [36]. All these results suggest that alterations in GABA levels are related to the onset of schizophrenia but depend on the specific brain region and medication [27, 36]. Our findings demonstrating reduced medial prefrontal GABA levels in drug-naïve FES patients provide more evidence that GABA alterations have an important impact on the early stages of psychosis.

Deficits in the GABA neurotransmitter system in schizophrenia have also been proven by postmortem and electrophysiological studies [57–59]. Postmortem studies have demonstrated GABA deficits in a subclass of fast-spiking interneurons expressing Ca^2+^-binding protein parvalbumin in patients with schizophrenia [60]. Reduced message RNA and expression of GAD67 (the synthetic enzyme for GABA) and GAT1 (the transporter that clears synaptic GABA) [58] as well as an apparent compensatory upregulation in postsynaptic GABA_A_ receptors in the dorsolateral prefrontal cortex [57, 61], ACC [58], and prefrontal cortex [45, 59, 62] have also been reported in chronic patients with schizophrenia. Electrophysiological studies have provided more evidence for the dysfunctional action of GABAergic transmission in schizophrenia reflected by deficits in gamma oscillation, P50, and the cortical inhibition index [63–67]. Attenuated gamma oscillation has been observed in FES patients [66]. Reduced auditory P50 suppression has reflected disrupted inhibitory gating of the brain in response to repeated auditory stimuli in drug-naïve FES patients [63, 65]. FES patients showed reduced short-interval cortical inhibition (SICI) and a prolonged cortical silence period (CSP) compared with healthy control subjects, suggesting weakened GABA_A_-mediated inhibition [64, 66]. All these findings further support the hypothesis that dysfunctional GABAergic transmission exists in FES patients [63–67].

In the present study, low medial prefrontal GABA levels were only observed in FES patients not in UHR subjects. A positron emission tomography (PET) study demonstrated the reduced binding potential of GABA_A_/benzodiazepine receptors in the right caudate in UHR individuals [68]. A recent MRS study indicated an elevated GABA/water ratio in the mPFC in UHR subjects [17]. In our previous study, we found that GABA_B_-mediated cortical inhibition was impaired in UHR, whereas GABA_A_-mediated cortical inhibition was not altered yet [69]. Whether GABA dysfunction has occurred before the onset of psychosis remains unclear. There was interplay between two GABA-mediated neurotransmitter systems (GABA_A_-mediated and GABA_B_-mediated ones) [70, 71]. Thus, there might be a compensatory effect between two GABA subsystems, leading to GABA deficits in FES patients rather than UHR subjects [64, 69]. Previous studies indicated that FES patients showed a reduced SICI and a prolonged duration of CSP, implying both GABA_A_ and GABA_B_ dysfunction, whereas UHR subjects only had a reduced SICI or prolonged duration of CSP, suggesting partially impaired GABA functions [64, 69]. Thus, medial prefrontal GABA reductions could be a candidate biomarker for the early stages of psychosis.

In addition, we found significant correlations between Glx and GABA levels in FES patients, in accordance with previous studies [17, 19]. Kegeles et al. reported a positive correlation between GABA and Glx levels in the mPFC in medicated and unmedicated patients with schizophrenia and HC subjects [19]. de la Fuente-sandoval et al. found a positive correlation between GABA and Glx levels across UHR and HC subjects [17]. Glu serves as a precursor for the synthesis of GABA and glutathione [5–8]. We hypothesized that the reduction of GABA levels might be secondary to a reduction in the glutamatergic system. There was also a significant correlation between GABA and Glx levels in UHR subjects, and both metabolites remained unchanged. Natsubori et al. also reported unchanged mPFC Glx levels in UHR subjects [30]. These findings suggest that the interplay of GABA and Glx systems might act in a mutually compensatory manner. Both impaired GABAergic and glutamatergic systems may contribute to the development of psychosis [25, 27].

There are limitations of the present study; thus, we should be cautious when interpreting our results. First, MRS data were only acquired in one region (mPFC), which made our results less comparable with previous studies on other regions, for example, the striatum, which will be improved in our further work. Second, we only recruited drug-naïve subjects, which made our sample size for each group relatively small. Considering that the heterogeneity of FES patients and UHR subjects may affect metabolic levels, further studies with a large sample size will be helpful in confirming our findings.

## 5. Conclusions

In summary, we found both reduced medial prefrontal GABA and Glx concentrations only in drug-naïve FES patients but not in drug-naïve UHR subjects, suggesting that GABA and Glx alterations may be associated with the early stages of psychosis.

## Figures and Tables

**Table 1:** Demographic and clinical characteristics among patients with first-episode schizophrenia (FES), subjects at ultrahigh risk for psychosis (UHR), and healthy controls (HC).

	HC	UHR	FES	Statistical significance
Group size	23	21	16	—
Age (years)	22.52 (5.50)	21.05 (5.69)	22.13 (5.49)	*F*(2.57) = 0.40, *P* = 0.67
Years of education	11.73 (2.03)	10.90 (2.17)	12.31 (2.47)	*F*(2.57) = 2.00, *P* = 0.15
Gender (male/female)	11/12	12/9	8/8	*χ* ^2^ = 0.41, *P* = 0.82
Handedness (right/left)	23/0	21/0	16/0	—
Tobacco use	4/23	0/21	0/16	*χ* ^2^ = 8.14, *P* = 0.017
SIPS/SOPS				
Positive	—	9.10 (3.00)	—	
Negative	—	12.48 (5.33)	—	
General	—	7.67 (2.97)	—	
Disorganization	—	9.29 (3.39)	—	
BPRS				
Positive	—	—	12.19 (3.75)	
Negative	—	—	8.38 (2.53)	
Total	—	—	43.56 (4.15)	

SIPS/SOPS: Structured Interview for Prodromal Syndromes and Scale of Prodromal Syndromes; BPRS: Brief Psychiatric Rating Scale.

**Table 2:** Means (SD) for tissue composition and spectral quality in the mPFC region in first-episode schizophrenia (FES), subjects at ultrahigh risk for psychosis (UHR), and healthy controls (HC).

Group	Tissue composition			Spectral quality	
	CSF fraction	GM fraction	WM fraction	FWHM (ppm)	SNR
HC	0.12 (0.030)	0.56 (0.032)	0.32 (0.049)	0.11 (0.04)	22.43 (5.13)
UHR	0.12 (0.028)	0.56 (0.038)	0.31 (0.046)	0.11 (0.03)	21.90 (4.29)
FES	0.11 (0.027)	0.56 (0.040)	0.32 (0.047)	0.11 (0.02)	20.81 (3.12)

GM: grey matter; WM: white matter; CSF: cerebrospinal fluid; FWHM: full-width at half maximum; SNR: signal-to-noise ratios.
